# Accurate bacterial outbreak tracing with Oxford Nanopore sequencing and reduction of methylation-induced errors

**DOI:** 10.1101/gr.278848.123

**Published:** 2024-11

**Authors:** Mara Lohde, Gabriel E. Wagner, Johanna Dabernig-Heinz, Adrian Viehweger, Sascha D. Braun, Stefan Monecke, Celia Diezel, Claudia Stein, Mike Marquet, Ralf Ehricht, Mathias W. Pletz, Christian Brandt

**Affiliations:** 1 Institute for Infectious Diseases and Infection Control, Jena University Hospital, 07747 Jena, Germany;; 2 Diagnostic and Research Institute of Hygiene, Microbiology and Environmental Medicine, Medical University of Graz, 8010 Graz, Austria;; 3 Institute of Medical Microbiology and Virology, University Hospital Leipzig, 04103 Leipzig, Germany;; 4 InfectoGnostics Research Campus, Center for Applied Research, 07743 Jena, Germany;; 5 Leibniz-Institute of Photonic Technology (Leibniz-IPHT), 07745 Jena, Germany;; 6 Institute of Physical Chemistry, Friedrich-Schiller-University Jena, 07743 Jena, Germany

## Abstract

Our study investigates the effectiveness of Oxford Nanopore Technologies for accurate outbreak tracing by resequencing 33 isolates of a 3-year-long *Klebsiella pneumoniae* outbreak with Illumina short-read sequencing data as the point of reference. We detect considerable base errors through cgMLST and phylogenetic analysis of genomes sequenced with Oxford Nanopore Technologies, leading to the false exclusion of some outbreak-related strains from the outbreak cluster. Nearby methylation sites cause these errors and can also be found in other species besides *K. pneumoniae*. Based on these data, we explore PCR-based sequencing and a masking strategy, which both successfully address these inaccuracies and ensure accurate outbreak tracing. We offer our masking strategy as a bioinformatic workflow (MPOA) to identify and mask problematic genome positions in a reference-free manner. Our research highlights limitations in using Oxford Nanopore Technologies for sequencing prokaryotic organisms, especially for investigating outbreaks. For time-critical projects that cannot wait for further technological developments by Oxford Nanopore Technologies, our study recommends either using PCR-based sequencing or using our provided bioinformatic workflow. We advise that read mapping–based quality control of genomes should be provided when publishing results.

Whole-genome sequencing is essential for analyzing outbreaks, pandemics, or phylogenetic relationships (Chewapreecha et al. 2017; Wyres et al. 2020). The recent SARS-CoV-2 pandemic has thus led to a leap in the integration and expansion of sequencing capacities in many laboratories and hospitals, predominantly using Illumina for short-read sequencing or Oxford Nanopore Technologies (ONT) for long-read sequencing (∼78% and 18%, respectively) (Brandt et al. 2021). Beyond viral pandemic tracking, bacterial pathogen outbreaks, particularly those linked to antibiotic resistance, continue to impose a significant global public health burden (Murray et al. 2022). Gram-negative bacteria, in particular, rapidly acquire antibiotic resistance via horizontal gene transfer from other species (Lerminiaux and Cameron 2019; Hadjadj et al. 2022; Moura de Sousa et al. 2023). This mechanism complicates tracking outbreaks or identifying their origin, as a single specific plasmid or mobile element can be responsible for a persistent outbreak or multiple outbreaks across unrelated species (Sivertsen et al. 2014; Pletz et al. 2018; Abe et al. 2021; Hadjadj et al. 2022). Additionally, in Gram-negative bacteria, DNA methylation plays a crucial role in epigenetic regulation, which impacts gene expression, genome modification, virulence, mismatch repair, and other physiological activities (Gao et al. 2023; Wang et al. 2023).

Effectively tracking these complex molecular mechanisms requires careful strategic monitoring and sequencing-based investigation. Consequently, the accuracy and continuity of the genome data are paramount. Illumina, a short-read sequencing method with an error rate of <0.8% in raw data, is frequently used as its complementary genome reconstruction precision exceeds 99.997% (Wang et al. 2021). However, repetitive elements, such as transposons, present a substantial challenge for short reads when reconstructing closed bacterial genomes and their accompanying plasmids. Long-read sequencing technologies like Pacific Biosciences (PacBio) and ONT can resolve such elements, for example, plasmids, as they achieve longer read lengths averaging ∼10–20 kb and even up to 3.85 Mb in the case of ONT (Eid et al. 2009; Grohme et al. 2018; Tyson et al. 2018; Dohm et al. 2020).

Real-time sequencing allows data collection and analysis, whereas sequencing positions ONT as an appealing choice for hospital surveillance and outbreak control (Spott et al. 2022). Owing to their recently launched flow cells (R10.4.1) and chemistry (SQK-NBD114.24), they have achieved raw read accuracy that now exceeds 99.1% (Ni et al. 2023). Several studies have shared their findings and reported accuracy levels similar to those from short-read data (Sanderson et al. 2023; Wagner et al. 2023). However, significant discrepancies between Illumina and ONT genomes were also observed for some organisms (Linde et al. 2023).

These contradictions can lead to inaccurate conclusions, like excluding outbreak-associated samples when investigating outbreaks. In addition, genomes are usually stored in open public databases such as NCBI or ENA, which can lead to error propagation and can potentially significantly affect patients’ welfare. Therefore, we used ONT to reevaluate a well-documented, 3-year-long outbreak initially analyzed with Illumina data to address these contradictory statements, focusing on the errors in ONT sequencing data (Viehweger et al. 2021). *Klebsiella pneumoniae* is an ideal microorganism for this topic, as it is a common pathogen linked to hospital-wide outbreaks carrying plasmids with multidrug-resistance genes (Brandt et al. 2019). When using ONT-only data, we identified a few critical issues leading to erroneous basecalls for *K. pneumoniae*. We noticed similar problems and clear patterns in other organisms, which need to be considered during outbreak identification, even though we could resolve them.

## Results

### Erroneous basecalls occur in some strains but not others and vary by basecaller and sequencing kits

**Figure 1.**
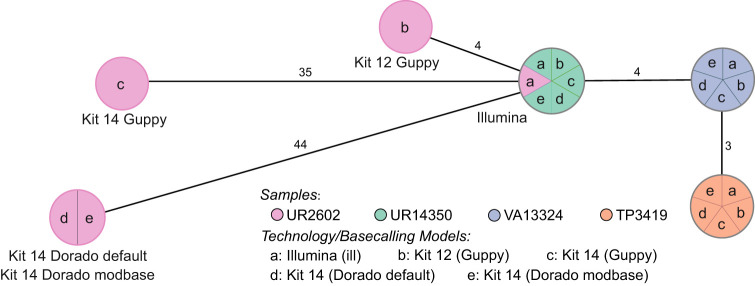
cgMLST typing reveals allelic differences between genomes utilizing different basecaller models and sequencing kits. The minimum spanning tree pictures four *K. pneumoniae* samples based on 2358 genes for pairwise comparison of allelic variations. Missing values were ignored. Nodes (samples) are connected by lines depicting the distance by numbers of allelic differences. Loci are considered different if one or more bases change between the samples. Loci without allelic differences are described as being the same. Samples with allelic differences of 15 or fewer are considered as part of the cluster. All isolates were prepared with Kit 14 and Kit 12 and basecalled with each respective Guppy “superaccurate” basecalling model (see subsection “Basecalling and assembly” in the Methods). We basecalled all Kit 14–prepared samples with Dorado using the default and a modification-aware model (see subsection “Basecalling and assembly” in the Methods).

We resequenced the genomes of 33 randomly distributed *K. pneumoniae* samples isolated from 31 patients (from a total of 114 outbreak-related isolates) using R10.4 and R10.4.1 flow cells, along with the corresponding library preparation kits (henceforth “Kit 12”: SQK-NBD112.24 [early access] and “Kit 14”: SQK-NBD114.24 [successor]). Our objective was to investigate whether previously reported conflicting statements could be replicated (Linde et al. 2023; Sanderson et al. 2023; Wagner et al. 2023). We used core genome multilocus sequence typing (cgMLST) to compare ONT and Illumina-sequenced genomes. The comparison of the 33 samples revealed 11 outliers in the ONT data, showing high allelic deviations (up to 46) to their short-read counterparts while not matching the outbreak cluster (Supplemental Figs. 1 and 2). Although the remaining samples closely match the outbreak cluster, the outliers highlight inconsistencies within the ONT data, as reported in the literature. To assess whether either the basecaller or their models might be responsible, we re-basecalled and compared an outlier sample (UR2602) in detail to three samples, for which we assume error-free genomes based on cgMLST (for further details, see subsection “Basecalling and assembly” in the Methods) (Fig. 1) and pairwise SNP calling (Supplemental Fig. 3) with Illumina genomes.

Based on 2358 loci for the cgMLST, no allelic differences, regardless of kit, basecaller, or basecaller model, were identified for the isolates UR14350, VA13324, and TP3419. In contrast, the outlier sample (UR2602) revealed allelic variations for each kit, basecaller, and sequencing technology. Despite both basecallers using the same raw signal data, the 35 allelic variations in Guppy differ without accordance from the 44 allelic variations in Dorado.

By cgMLST, the outlier sample prepared with Kit 14 would be falsely assigned as not part of the outbreak owing to its 35 or 44 allelic differences, even though the sample exhibits low allelic differences according to the short-read data (gold standard). Conversely, when prepared with early access Kit 12, the same isolate would be correctly considered as only four different loci could be observed (adhering to the recommended allelic difference cutoff of 15 or less) (Miro et al. 2020). Because the basecalling models disagreed on the allelic differences, we suspected more issues within the raw data (reads and raw signals) and conducted a comprehensive analysis of all possible affected positions.

### Ambiguities in purine or pyrimidine discrimination for a subset of genome positions can cause erroneous basecalls

**Figure 2.**
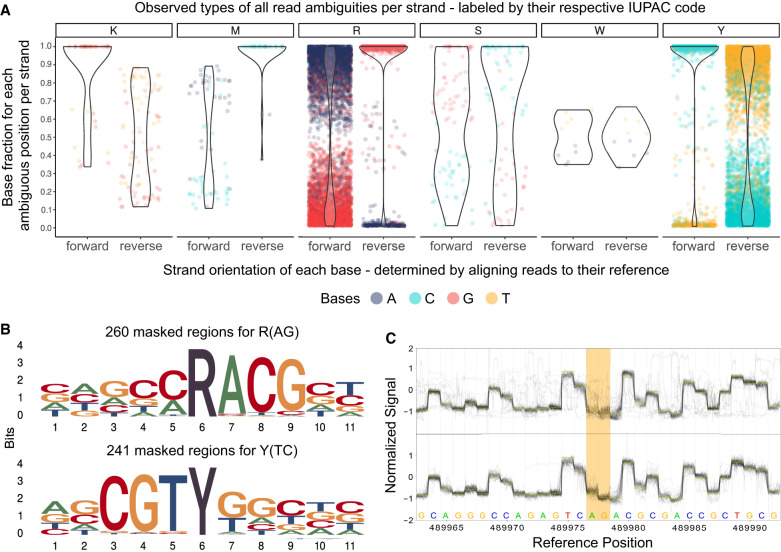
Systematic examination of ambiguous positions for frequency by strand orientation (*A*), conserved sequences (*B*), and raw Nanopore signal (*C*). (*A*) Violin chart showing the ratio between two bases within the mapped read data separated by strand orientation for 6556 ambiguous positions in 33 *K. pneumoniae* samples. Every ambiguous position is divided by which two bases appear and is labeled by their respective degenerative base (IUPAC nucleotide code). For example, “R” stands for a combination in which either A or G is found at that position. Each dot represents a base occurrence within the respective base combination at the ambiguous position. (*B*) Sequence logo of observed sequence pattern around the ambiguous bases R and Y on the chromosomal contig of *K. pneumoniae* for one sample. (*C*) Raw signal level (FAST5/POD5) of ambiguous positions (yellow) for Kit 14 (*above*) with methylated bases and SQK-RPB114.24 without modifications (*below*). Less clear signals are observable in ambiguous positions (yellow) for Kit 14. Signal plots were generated with remora (v.2.1.3; https://github.com/nanoporetech/remora).

The first visual inspection of mapped reads to the assembly revealed read ambiguity on certain positions indicated by varying base ratios (ambiguous positions). For further characterization of these ambiguous positions, we examined our data at the sequence, nucleotide, and raw signal level (Fig. 2). For each ambiguous position on the chromosomal DNA for 33 *K. pneumoniae* outbreak samples, we determined the ratio between the two bases by counting their occurrences for both strand orientations within the read data at that position (Fig. 2A). Searching for characteristic “indicator” sequence motifs, we explored the surrounding base for each detected ambiguous position and plotted the observed pattern as a sequence logo (Fig. 2B; Supplemental Table 1). Additionally, we compared the methylated and unmethylated raw signals around these ambiguous positions (Fig. 2C).

As highlighted in Figure 2A, in some positions, the basecaller cannot determine between either of two bases, expressed by specific base ratios varying per position and strand orientation, resulting in erroneous assemblies. We could not observe ambiguous positions containing more than two different bases. For clarity, we assign the IUPAC nucleotide code for degenerate bases (R, Y, M, K, S, W) to each ambiguous position varying between two bases. Accordingly, we will refer, for example, to “R” when the positions contain A or G in the read data.

Out of our analysis of 33 *K. pneumoniae* outbreak isolates, we discovered 6556 positions that exhibit ambiguity (Fig. 2A). The ambiguity mainly resolved around 3311 positions for R and 3111 for Y. In 5442 of 6455 R and Y positions (84.31%), the basecalled reads lean toward cytosine or guanine (C or G). We detected other ambiguous positions in K (44), M (34), S (51), and W (5), but with comparable lower occurrences. It is essential to acknowledge that not all identified ambiguous positions result in errors in the assembled genome, which explains the varying error profile of the same sample (Fig. 1). Errors in these ambiguous positions mainly arise when deciding between purine bases (A or G) or pyrimidine bases (T or C). Because strand bias is reported as a substantial factor for false-positive variant detection, we considered the strand orientation of the read data (Leija-Salazar et al. 2019). In most cases, we noticed that the correct base is located clearly and more frequently on both strand orientations, whereas the incorrect base is less prevalent. For instance, the correct base guanine is found on both strands for positions masked by R and is predominantly found on the reverse strand (Fig. 2A, see R reverse) and less frequently on the forward strand, which also contains the false base. We detected similar behavior for other species (Supplemental Fig. 6).

In the error-prone genomes of the *K. pneumoniae* outbreak, we detected preserved patterns around the ambiguous positions R and Y (Fig. 2B). These sequence motifs are reverse-complement patterns (RACG/CGTY), pointing to a singular issue. We also observed additional patterns compared with other isolates of *K. pneumoniae*. These motifs are likely specific to particular strains.

Furthermore, we examined and collected additional ONT sequencing data that used the Kit 14 library preparation (264 isolates across 32 species) to investigate whether the ambiguous positions are *K. pneumoniae* exclusive (Table 1). These samples were collected based solely on Kit 14 library preparations and not on whether they were associated with an outbreak. We determine, compared to all other species samples, the fewest ambiguous positions (zero to one) in *Bordetella pertussis*. In contrast, all 10 *Enterococcus faecalis* isolates had more than 200 ambiguous positions. More than 40% of 264 screened samples have more than 50 ambiguous positions. Across all species, the minimal shared sequence motif was RA/TY. Certain species, such as *Acinetobacter junii*, *Acinetobacter radioresistens*, *Chryseobacterium gleum*, *Enterobacter cloacae*, *Micrococcus luteus*, and *Stenotrophomonas maltophilia*, exhibited a considerable number of ambiguous positions. This suggests that many species may be impacted, but not necessarily all strains.

**Table 1.** Overview of R (A – G) and Y (T – C) base ambiguity for 264 isolates from 32 species, sequenced with Oxford Nanopore Technologies using Kit 14

Species	Total samples	Samples with >50 ambig. P	Mean R (A – G) ambiguity per sample (min/max)	Mean Y (T – C) ambiguity per sample (min/max)	Motif type	Reference
*Achromobacter xylosoxidans*	1	0 (0%)	6	4	NA	^a^
*Acinetobacter baumannii*	15	5 (33.33%)	22.53 (0/109)	19.87 (0/83)	NA	^a^
*Acinetobacter junii*	1	1 (100%)	279	223	CA**R**ATG CAT**Y**TG	^a^
*Acinetobacter mesopotamicus*	1	1 (100%)	53	28	NA	^a^
*Acinetobacter radioresistens*	1	1 (100%)	175	150	**R**A T**Y**	^a^
*Acinetobacter soli*	1	0 (0%)	12	11	NA	^a^
*Bordetella pertussis* ^d^	40	0 (0%)	0.1 (0/1)	0.2 (0/1)	NA	(Wagner et al. 2023)
*Chryseobacterium arthrosphaerae*	1	0 (0%)	13	7	NA	^a^
*Chryseobacterium gleum*	1	1 (100%)	218	217	**R**ACGC GCGT**Y**	^a^
*Citrobacter freundii*	3	3 (100%)	145.67 (49/329)	137.67 (39/319)	C**R**ATGTCGACAT**Y**G	^a^
*Citrobacter portucalensis*	2	2 (100%)	29 (26/32)	29 (28/30)	**R**A T**Y**	^a^
*Enterobacter cloacae*	1	1 (100%)	153	162	NA	^a^
*Enterobacter hormaechei*	5	1 (20%)	15.60 (4/45)	15.60 (6/42)	NA	^a^
*Enterococcus faecalis*	10	10 (100%)	250.40 (223/275)	249.00 (210/270)	T**R**AG CT**Y**A	^c^
*Enterococcus faecium*	19	2 (10.53%)	15.74 (0/27)	14.63 (0/28)	**R**ACC GGT**Y**	(Dabernig-Heinz et al. 2024)^b^
*Escherichia coli*	8	3 (37.50%)	31.38 (2/118)	29.31 (4/111)	NA	^a^
*Escherichia flexneri*	10	9 (90%)	49.10 (19/88)	44.70 (18/88)	**R**AT AT**Y**	^a^
*Klebsiella aerogenes*	1	0 (0%)	22	17	NA	^a^
*Klebsiella michiganensis*	1	1 (100%)	56	42	NA	^a^
*Klebsiella pneumoniae*	70	38 (54.29%)	97.04 (3/835)	92.47 (3/847)	**R**ACG CGT**Y**	(Viehweger et al. 2021)^a,b^
*Klebsiella oxytoca*	1	0 (0%)	5	9	NA	^a^
*Listeria monocytogenes*	17	3 (17.65%)	37.94 (0/514)	40.82 (0/557)	NA	^b^
*Micrococcus luteus*	1	1 (100%)	172	220	C**R**AC GT**Y**G	^a^
*Proteus mirabilis*	1	1 (100%)	45	43	C**R**AC GT**Y**G	^a^
*Pseudomonas aeruginosa*	19	6 (33.33%)	389.17 (0/2251)	387.56 (0/2290)	AA**R**ACC GGT**Y**TT	^a^
*Pseudomonas asiatica*	2	1 (50%)	145 (0/290)	144.50 (0/289)	CC**R**A T**Y**GG	^a^
*Pseudomonas stutzeri*	1	0 (0%)	14	16	NA	^a^
*Salmonella enterica*	2	1 (50%)	41.5 (7/76)	42.5 (7/78)	NA	^a^
*Stenotrophomonas maltophilia*	1	1 (100%)	229	171	TAC**R**AC GT**Y**GTA	^a^
*Serratia marcescens*	4	4 (100%)	107.75 (68/178)	99.5 (67/171)	CC**R**A T**Y**GG	^a^
*Shewanella algae*	2	2 (100%)	71.5 (37/106)	50 (32/68)	NA	^a^
*Staphylococcus aureus*	20	8 (40%)	23.35 (0/99)	23.35 (0/97)	**R**ACC GGT**Y**	^b^

Only chromosomal contigs were analyzed, and only “superaccurate” basecalling models were used. Chromosomes were coverage-masked by N if below a read depth of 10×. N positions were not considered for the table to avoid overestimating one base ambiguity.

^a^Sequenced strains received from Leibniz-Institute of Photonic Technology, Optisch-Molekulare Diagnostik und Systemtechnologie.

^b^Sequenced strains received from Diagnostic and Research Institute of Hygiene, Microbiology and Environmental Medicine, Medical University of Graz.

^c^Own samples from the Jena University Hospital.

^d^Sequenced with Kit 12; resequencing via Kit 14 showed no differences.

As methylated bases are probably liable for ambiguous positions, we compared sequencing data with methylations (Kit 14) (Fig. 2C, above) and without (SQK-RPB114.24) (Fig. 2C, below) on the raw signal level from FAST5/POD5 files before basecalling occurs. We choose the raw signal level for investigation prior to any bioinformatic approaches (e.g., basecalling, assembly, polishing) to avoid the potential introduction of other biases or errors. For native sequencing, less clear signals at these positions are observable, which might cause these ambiguous basecalls. These noisy signals could explain the frequencies of bases we detected in the reads (Fig. 2A) and, thus, the basecaller's difficulty in deciding on a specific base for that position.

We found no coherent methylation motifs in the literature that would fit the observed pattern. Nevertheless, it has been reported that methylated bases can affect the raw signal in the surrounding region (Tourancheau et al. 2021). Thus, we cannot determine whether multiple methylation motifs are the cause or whether an unknown motif is present. Accordingly, to these findings, we evaluated whether PCR-based sequencing or a bioinformatic masking strategy for ambiguous positions can reliably remove these methylation-based errors for outbreak analysis.

### Strategies to mitigate methylation-induced basecalling errors

**Figure 3.**
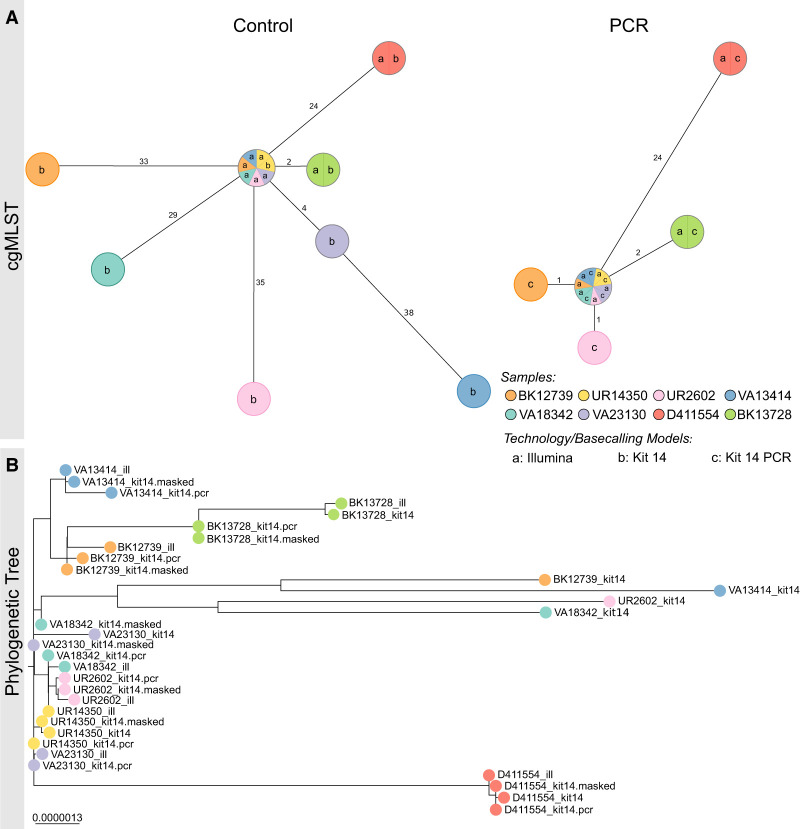
PCR-based sequencing or masking of ambiguous positions reduces allelic or phylogenetic distances. (*A*) Minimum spanning trees (pairwise ignore missing values) of each of eight *K. pneumoniae* outbreak samples based on 2358 genes to compare allelic differences between Illumina and Nanopore SQK-NBD114.24 genomes (Kit 14; *left*) and Illumina and Nanopore SQK-RPB114.24 genomes (PCR; *right*). Nodes (samples) are connected by lines depicting the distance by numbers of allelic differences. Loci are considered different whether one or more bases change between the samples. Loci without allelic differences are described as being the same. Samples with allelic differences of 15 or fewer are considered as part of the cluster. (*B*) Phylogenetic tree based on core genome SNP alignment between eight *K. pneumoniae* outbreak samples (colored nodes), prepared with Illumina (ill), Nanopore SQK-NBD114.24 (Kit 14), and SQK-RPB114.24 (Kit 14 PCR) compared with the masked Kit 14 assemblies (masked).

To solve methylation-induced basecalling errors in ambiguous base positions, we evaluated two strategies: (1) We resequenced 10 *K. pneumoniae* outbreak samples using the Nanopore rapid PCR barcoding kit (SQK-RPB114.24) to remove methylated bases prior to sequencing and analyzed the genomes using cgMLST and phylogenetic analysis (Fig. 3A,B), and (2) we masked ambiguous positions for Kit 14 prepared genomes with our bioinformatic workflow (see subsection “Workflow for detection and masking of ambiguous positions” in the Methods). It is important to mention that these masked assemblies cannot be used for cgMLST because allelic differences cannot be accurately determined for genes with masked bases. Therefore, the masked genomes were only used for phylogenetic analysis (Fig. 3B). Furthermore, it should be noted that cgMLST only considers coding sequences, whereas the phylogenetic analysis assesses the entire genome that is represented in all samples, which gives a higher resolution for base differences.

When comparing the native barcoding with the PCR-based kit, ambiguous positions were significantly reduced from 2357 to just 44 for R and Y across the 10 resequenced *K. pneumoniae* samples (Supplemental Table 2). Both the minimum spanning trees and the phylogenetic tree also show this significant improvement in genome quality for ONT (Fig. 3). According to the cgMLST, the outlier samples UR2602 and BK12739 now closely match the Illumina genome, down to only one allele difference, from 35/33 (Fig. 3A). When comparing phylogenetic distances within the phylogenetic tree, an increased convergence with the Illumina genomes, particularly for the outlier samples, was observed, too (Fig. 3B). Additionally, masked and PCR-based assemblies have almost no phylogenetic divergence.

Further, we analyzed the phylogenetic tree containing native Kit 14, masked native Kit 14, and Illumina genomes for all 33 *K. pneumoniae* samples (Supplemental Fig. 4). These include 11 Kit 14 outliers (average of 492 ambiguous positions) and 22 Kit 14 genomes with an average of less than 52 ambiguous positions. We observed two types of phylogenetic distances between ONT Kit 14 and Illumina: The expected considerable distances between the outlier and Illumina genomes are because of ambiguity and, in some cases, a phylogenetic distance for which ambiguity is not the causation.

By masking ambiguous bases, we observed that eight of 11 outlier genomes now closely align with their respective Illumina genome. The remaining three outlier samples changed their tree positions after masking, now closely aligned with other Illumina genomes but still diverged from their corresponding Illumina genome owing to other non-ambiguity-related differences. For the other 22 masked Kit 14 genomes with less ambiguity than the outliers, we did not observe any substantial changes in their tree positions, as fewer positions were masked.

In summary, 22 out of 33 masked ONT genomes align with their respective Illumina genomes, and the remaining 11 do not. In these cases, the remaining distances do not result from ambiguous positions within the ONT assemblies. For instance, the Illumina and ONT genomes of TP3870 matched perfectly in the minimum spanning tree, but they exhibited some distance from each other in the phylogenetic tree (Supplemental Fig. 4). We identified reconstruction issues in these short-read assemblies, primarily manifesting in noncoding regions (Supplemental Fig. 5). Because cgMLST only compares coding sequences, these errors do not affect the result analysis. Therefore, we recommend using only one technology when performing whole-genome comparison for outbreak analysis.

## Discussion

Over the past few years, ONT has been effectively used to monitor and track the SARS-CoV-2 pandemic and its viral lineages. Despite this, contradictory reports have emerged regarding the consistency of ONT-sequenced bacterial genomes compared with those that are Illumina based. Our research examined whether ONT can be used to analyze bacterial outbreaks accurately.

For our investigation, we resequenced a well-documented, 3-year-long *K. pneumoniae* outbreak using the Nanopore native barcoding Kit 14 for library preparation. Our analyses demonstrated that the raw signals were impacted by methylated bases, creating ambiguous positions through basecalling and leading to erroneous exclusions of certain outbreak-associated strains. However, not all isolates are affected by these ambiguities, and none or minimal allelic differences are shown in the corresponding short-read data. One should note that some errors in noncoding areas for Illumina assemblies were observed, which can lead to higher distances within a phylogenetic tree but with little to no effect in cgMLST. Despite focusing on *K. pneumoniae* initially, other prokaryotic organisms are also impacted. Crucially, we also detected ambiguous positions using the predecessor sequencing kit SQK-LSK109 (Supplemental Table 3). Consequently, when using ONT-based sequence data from open public databases or when analyzing outbreaks, one should test for read ambiguity by using, for example, the provided MPOA workflow before further analysis.

Based on our in-depth investigation, we recommend using the Nanopore rapid PCR barcoding kit for sequencing to eliminate these read ambiguities in the genome assemblies. However, this method decreases the read length to ∼3500 bp, posing difficulties in achieving closed plasmids and genomes, similar to other short-read approaches but to a way lesser extent. A higher sequencing depth might also be necessary to control for polymerase errors. For samples already sequenced without any involvement of PCR, we propose using the provided MPOA workflow to assess the quality of each genome. This workflow offers information about the frequency and strand orientation of reads in ambiguous positions and masks them in the assembly by the IUPAC nucleotide code without needing another reference. These masked assemblies can be used for constructing phylogenetic trees for outbreak tracking. However, cgMLST cannot be performed as masked or degenerated bases create false allelic differences across the whole minimal spanning tree.

Given the notable strides made in direct methylation calling techniques, ONT might overcome the issues with ambiguous positions. If available in high enough quantities, duplex reads (connecting and sequencing both strands) might provide better raw signal data for accurate basecalling. The recently introduced research model reduces the ambiguous positions in direct comparison, but still a few remain (research model “res_dna_r10.4.1_e8.2_400bps_sup” available at GitHub (https://github.com/nanoporetech/rerio); not yet implemented in MinKNOW version 23.07.12, as of November 30, 2023) (Supplemental Table 4). Further advances that reduce methylation-induced errors for certain specific motifs are being developed, as shown for *Listeria monocytogenes* and *Escherichia coli* (Hallgren et al. 2021; Chiou et al. 2023). Nevertheless, we strongly recommend constantly testing and evaluating reconstructed prokaryotic genomes to avoid erroneous conclusions based on these ambiguous positions introduced by unknown and not-yet-considered methylation motifs.

## Methods

### Isolates and genomic data

ONT sequencing data from three institutes have been collected and analyzed. The sequencing data include 264 isolates from 32 species, provided by the Leibniz-Institute of Photonic Technology Jena, Medical University of Graz, and University Hospital Jena. Additionally, a set of 80 samples containing *K. pneumoniae*, *E. faeces*, *L. monocytogenes*, and *Staphylococcus aureus* from a ring trail were used for analysis (Dabernig-Heinz et al. 2024). University Hospital Leipzig provided 33 carbapenem-resistant *K. pneumoniae* outbreak isolates from sequence type 258 and the associated Illumina sequencing data (Supplemental Table 5). We ensured these samples were scattered across the whole phylogenetic tree that Viehweger et al. (2021) provided in their paper.

### Genomic DNA isolation

Isolates from 10% glycerin cryo-culture were streaked out on Columbia agar with 5% sheep blood (Becton Dickinson). After overnight incubation, a single colony was selected and cultured overnight in MH-broth. Genomic DNA was isolated via ZymoBIOMICS DNA Microprep kit (ZymoResearch D4301 and D4305) with modifications to enhance the output yield. Qubit dsDNA BR assay-kit (Thermo Fisher Scientific) was employed to quantify DNA concentrations from each sample. This kit uses fluorescent dyes to measure double-stranded DNA to ensure reliable results.

### Whole-genome sequencing

To prepare the library for sequencing using ONT's GridION system, we used the native barcoding kit 24 V12 (ONT SQK-NBD112.24) and native barcoding kit 24 V14 (ONT SQK-NBD114.24) with R10.4 and R10.4.1 flow cells, respectively. Both sequencing protocols were optimized regarding prolonged incubation times. Additionally, one library was prepared with rapid PCR barcoding kit 24 (ONT SQK-RPB114.24) for sequencing on an R10.4.1 flow cell. Sequencing of libraries prepared with SQK-NBD112.24 and SQK-NBD114.24 was conducted at 4 kHz with 260 bp, whereas SQK-RPB114.24 was conducted at 5 kHz with 400 bp. The DNA fragments minimum length for all sequencing runs was set to 200 bp in MinKNOW (v22.12.5) software.

### Basecalling and assembly

Basecalling and barcode demultiplexing of long-read sequencing data were performed on the GridION (ONT) deploying Guppy (v6.4.6) using superaccurate models associated with the different used sequencing kits (“dna_r10.4_e8.1_sup.cfg,” “dna_r10.4.1_e8.2_260bps_sup.cfg,” “dna_r10.4.1_e8.2_5khz_400bps_sup.cfg”). For further analysis, Dorado (v0.3.0) was used (“dna_r10.4.1_e8.2_260bps_sup.cfg” and “dna_r10.4.1_e8.2_260bps_modbases_5mc_cg_sup.cfg”).

Reads were filtered by excluding them below 1000 bp with Filtlong (v.0.2.1) (https://github.com/rrwick/Filtlong). De novo assembly was conducted using Flye (‐‐meta ‐‐nano-hq; v2.9) (Kolmogorov et al. 2019). Filtered reads were mapped to the assembly using minimap2 (-ax map-ont; v2.18) (Li 2018) and polished afterward by Racon (v1.4.20; https://github.com/lbcb-sci/racon) followed by medaka_consensus polishing both in default settings (v1.5.0; https://github.com/nanoporetech/medaka) using the following models: *r104_e81_sup_g5015*, *dna_r10.4.1_e8.2_260bps_sup@v3.5.2*, *r1041_e82_260bps_sup_g632* (Supplemental Code 1). Short reads obtained by Illumina sequencing were assembled using Shovill (v1.1.0; https://github.com/tseemann/shovill), which includes various genome corrections and polishing approaches (Viehweger et al. 2021).

### cgMLST of *K. pneumonia*

For cgMLST, we used a species-specific public cgMLST scheme for a gene-by-gene comparison on an allelic level (Leopold et al. 2014; Mellmann et al. 2016) according to “*K. pneumoniae sensu lato* cgMLST” (https://www.cgmlst.org/ncs/schema/2187931/). This comprises a total of 2358 genes (∼40% of the NTUH-K2044 reference genome) (Bialek-Davenet et al. 2014) in Flye-assembled and polished long-read and Shovill-assembled short-read assemblies (v1.1.0; github.com/tseemann/shovill). To illustrate the clonal relationships between different isolates, a minimum-spanning tree analysis was performed based on the determined allelic profiles using the Ridom SeqSphere^+^ software version 7 (Ridom) (Jünemann et al. 2013) with the parameter “pairwise ignore missing values.” We defined a clonal transmission event if the isolates differ by 15 or more alleles for *K. pneumoniae* (Miro et al. 2020).

### Phylogenetic tree

The phylogenetic trees visualize the evolutionary relationship among *K. pneumoniae* outbreak strains and are constructed based on core genome SNP alignment using Snippy-core (v4.6.0; github.com/tseemann/snippy). The phylogenetic tree was built using FastTree (Price et al. 2009) and visualized in Microreact (Argimón et al. 2016).

### Workflow for detection and masking of ambiguous positions

**Figure 4.**
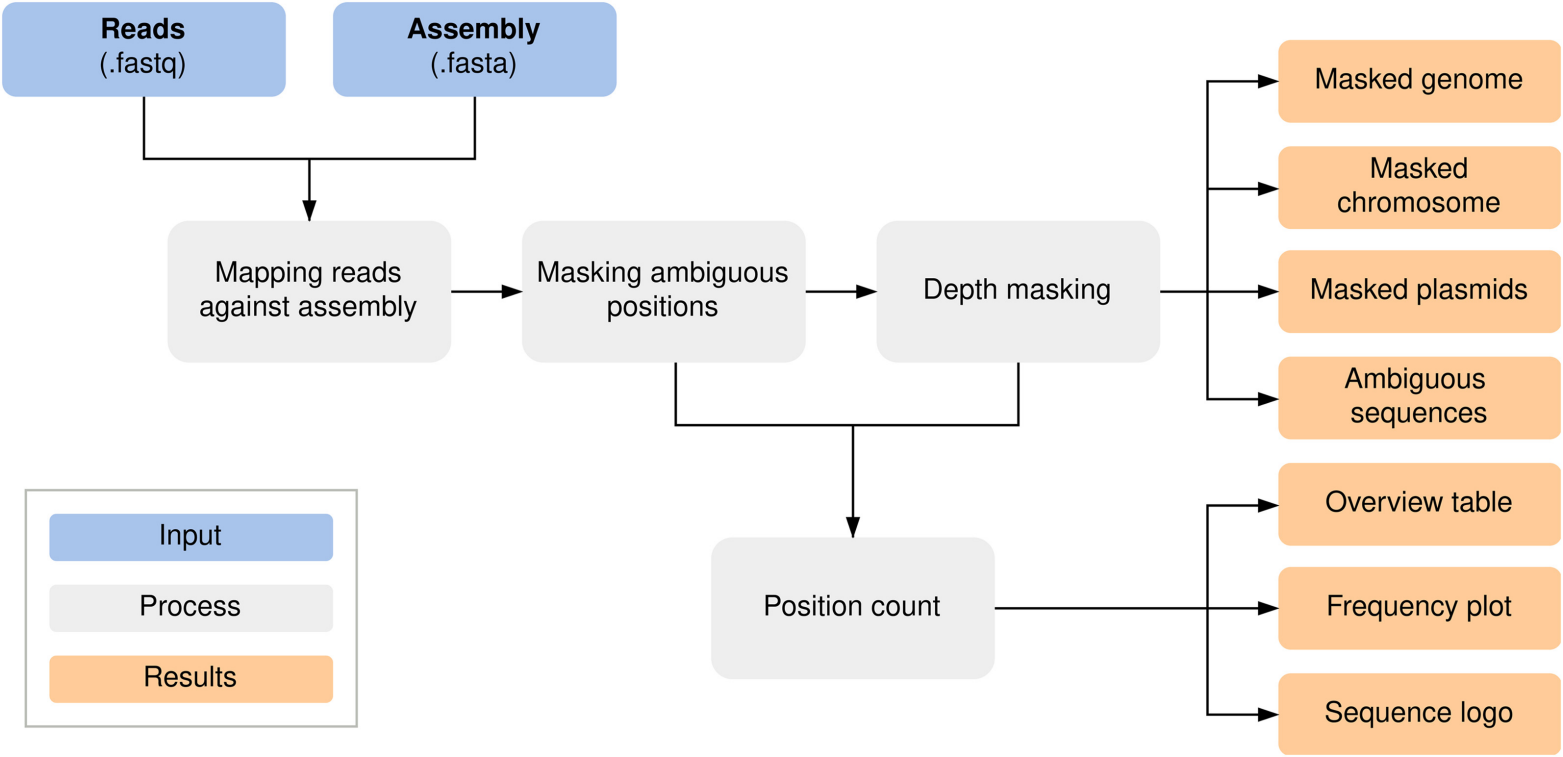
MPOA workflow to mask ambiguous and low coverage (0×–10× sequencing depth) positions in genome files. The workflow provides masked assemblies containing all contigs and separate masked chromosomes and plasmid FASTA files. A FASTA file per sample is also generated for each ambiguous position plus surrounding bases for further analysis (Supplemental Code 2; https://github.com/replikation/MPOA).

We developed a standardized Nextflow (Di Tommaso et al. 2017) workflow for de novo quality validation of all species, which is publicly available at GitHub (https://github.com/replikation/MPOA), licensed under GNU general public license v3.0. The workflow only needs the genome file (FASTA) and the associated reads (FASTQ) (Fig. 4). The workflow provides reproducible quality control by counting and summarizing ambiguous bases for the user, masking low coverage regions (0×–10× depth) with BEDTools (v2.31.0) (Quinlan and Hall 2010), and providing an assembly with these positions masked by the IUPAC nucleotide code for subsequent analysis. The workflow utilizes Docker or singularity container and is compatible with a local run, Slurm, or Google Cloud Compute. Identification and masking of ambiguous positions were conducted using SAMtools consensus (v1.17) (Li et al. 2009) after minimap2 (v.2.26; default) (Li 2018) or BWA (v.0.7.17; alternative) mapping (Li and Durbin 2010). Different mapping approaches were tested (Supplemental Figs. 7, 8). PlasFlow (v1.1.0) (Krawczyk et al. 2018) extracts chromosome contigs for downstream analysis without plasmid sequences. R was utilized to plot sequence motifs (Fig. 2B) with ggseqlogo (Wagih 2017) and a violin chart comparing base frequencies per strand (Fig. 2A) with ggplot2 (Wickham 2016).

## Data access

The ONT sequencing data generated in this study have been submitted to the NCBI BioProject database (https://www.ncbi.nlm.nih.gov/bioproject/) under accession number PRJNA1050168. The Illumina sequencing data used in this study have been submitted to the NCBI BioProject database (https://www.ncbi.nlm.nih.gov/bioproject/) under accession number PRJNA742413. The workflow used in this study has been uploaded to GitHub (https://github.com/replikation/MPOA) and as Supplemental Code 2.

## Supplemental Material

Supplement 1

Supplement 2

Supplement 3
