# Supplementary figures and images for: Accurate bacterial outbreak tracing with Oxford Nanopore sequencing and reduction of methylation-induced errors
Source: Genome Res. 2024 Nov;34(11):2039–47. doi: 10.1101/gr.278848.123 (PMC11610573; doi:10.1101/gr.278848.123)

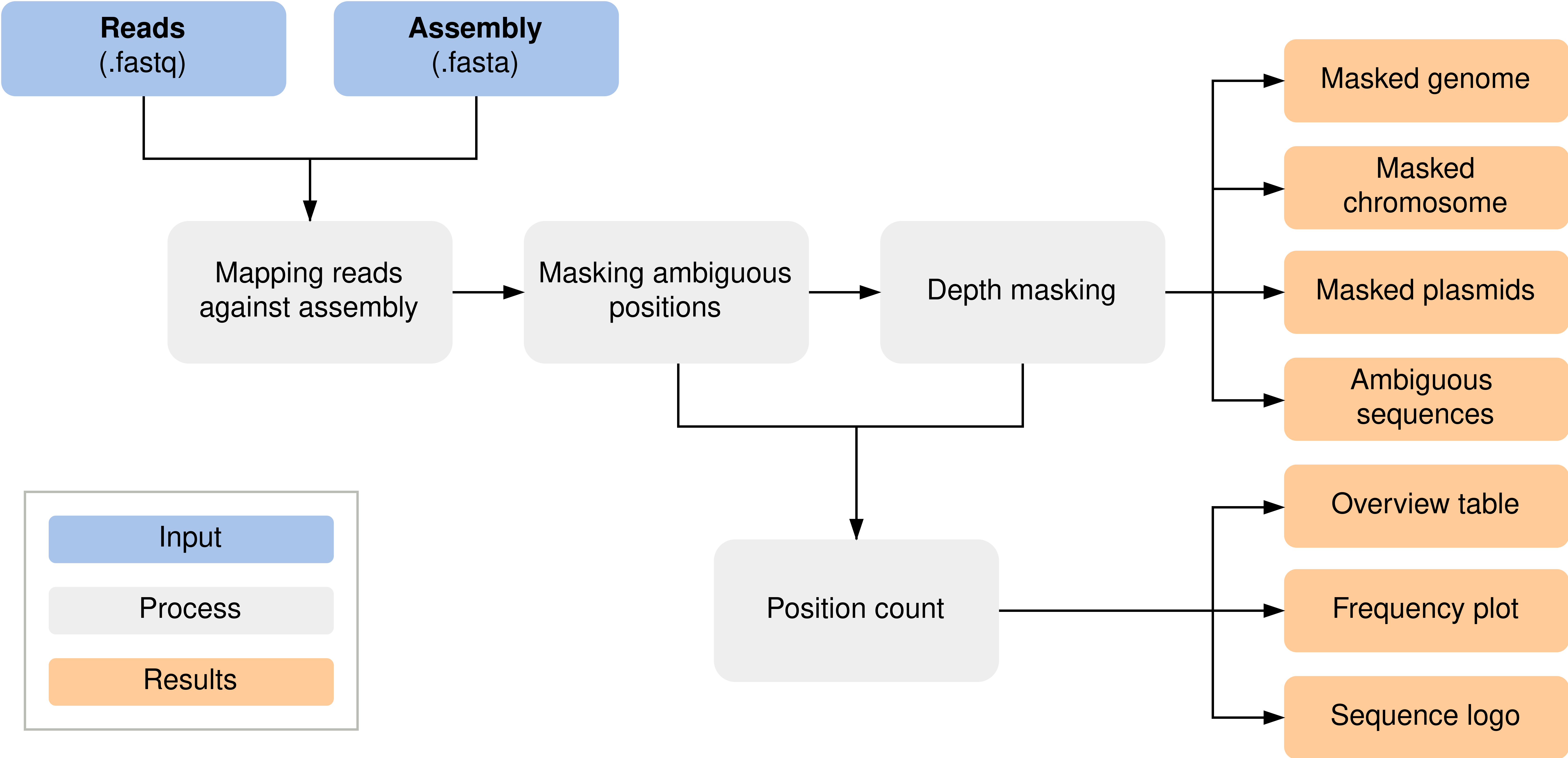

Supplement: Supplement 3 [file Supplemental_Code_2.zip › MPOA-1.4.2/data/figures/MPOA_flowchart.png]

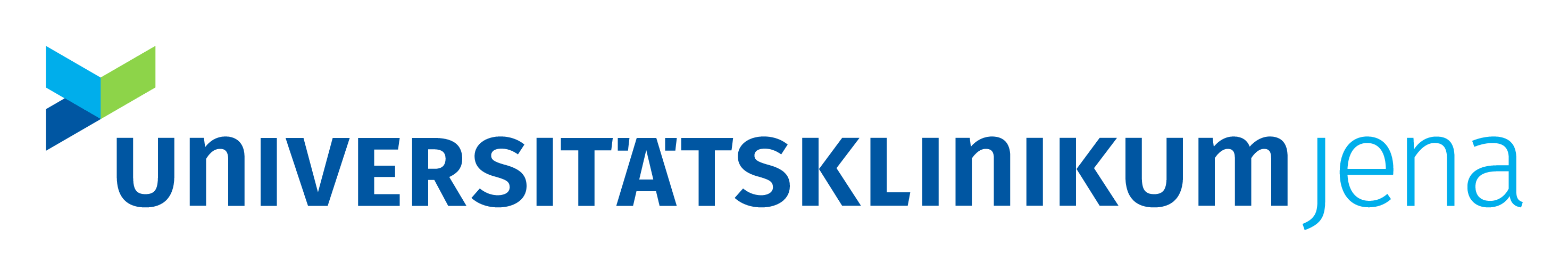

Supplement: Supplement 3 [file Supplemental_Code_2.zip › MPOA-1.4.2/data/logo/mobile_logo.png]
